# A generalized approach for producing, quantifying, and validating citizen science data from wildlife images

**DOI:** 10.1111/cobi.12695

**Published:** 2016-04-25

**Authors:** Alexandra Swanson, Margaret Kosmala, Chris Lintott, Craig Packer

**Affiliations:** ^1^ Department of Ecology, Evolution and BehaviorUniversity of MinnesotaSaint PaulMN 55108U.S.A.; ^2^ Department of PhysicsUniversity of OxfordDenys Wilkinson BuildingOxfordOX1 3RHU.K.; ^3^ Current address: Department of Organismic and Evolutionary BiologyHarvard UniversityCambridgeMA 02138U.S.A.

**Keywords:** big data, camera traps, crowdsourcing, data aggregation, data validation, image processing, Snapshot Serengeti, Zooniverse, cámaras trampa, conjunto de datos, crowdsourcing, datos grandes, procesamiento de imágenes, Snapshot Serengeti, validación de datos, Zooniverse

## Abstract

Citizen science has the potential to expand the scope and scale of research in ecology and conservation, but many professional researchers remain skeptical of data produced by nonexperts. We devised an approach for producing accurate, reliable data from untrained, nonexpert volunteers. On the citizen science website www.snapshotserengeti.org, more than 28,000 volunteers classified 1.51 million images taken in a large‐scale camera‐trap survey in Serengeti National Park, Tanzania. Each image was circulated to, on average, 27 volunteers, and their classifications were aggregated using a simple plurality algorithm. We validated the aggregated answers against a data set of 3829 images verified by experts and calculated 3 certainty metrics—level of agreement among classifications (evenness), fraction of classifications supporting the aggregated answer (fraction support), and fraction of classifiers who reported “nothing here” for an image that was ultimately classified as containing an animal (fraction blank)—to measure confidence that an aggregated answer was correct. Overall, aggregated volunteer answers agreed with the expert‐verified data on 98% of images, but accuracy differed by species commonness such that rare species had higher rates of false positives and false negatives. Easily calculated analysis of variance and post‐hoc Tukey tests indicated that the certainty metrics were significant indicators of whether each image was correctly classified or classifiable. Thus, the certainty metrics can be used to identify images for expert review. Bootstrapping analyses further indicated that 90% of images were correctly classified with just 5 volunteers per image. Species classifications based on the plurality vote of multiple citizen scientists can provide a reliable foundation for large‐scale monitoring of African wildlife.

## Abstract (es)

Una Estrategia Generalizada para la Producción, Cuantificación y Validación de los Datos de Ciencia Ciudadana a partir de Imágenes de Vida Silvestre

**Resumen**

La ciencia ciudadana tiene el potencial de expandir el alcance y la escala de la investigación en la ecología y la conservación, pero muchos investigadores profesionales permanecen escépticos sobre los datos producidos por quienes no son expertos. Diseñamos una estrategia para generar datos precisos y fiables a partir de voluntarios no expertos y sin entrenamiento. En el sitio web de ciencia ciudadana www.snapshotserengeti.org más de 28, 000 voluntarios clasificaron 1.51 millón de imágenes que fueron tomadas en un censo a gran escala de cámaras trampa en el Parque Nacional Serengueti, Tanzania. Cada imagen llegó, en promedio, hasta 27 voluntarios, cuyas clasificaciones se conjuntaron mediante el uso de un algoritmo de pluralidad simple. Validamos el conjunto de respuestas frente a un juego de datos de 3, 829 imágenes verificadas por expertos y calculamos tres medidas de certeza: nivel de concordancia entre las clasificaciones (uniformidad), fracción de clasificaciones que apoyan al conjunto de respuestas (fracción de apoyo) y fracción de clasificadores que reportaron “nada aquí” en una imagen que al final se clasificó como que sí tenía un animal (fracción en blanco). Estas medidas se usaron para estimar la confianza de que un conjunto de respuestas estuviera en lo correcto. En general, el conjunto de respuestas de los voluntarios estuvo de acuerdo con los datos verificados por los expertos en un 98 % de las imágenes, pero la certeza varió según la preponderancia de la especie, de tal forma que las especies raras tuvieron una tasa más alta de falsos positivos y falsos negativos. El análisis de varianza calculado fácilmente y las pruebas post‐hoc de Tukey indicaron que las medidas de certeza fueron indicadores significativos de si cada imagen estuvo clasificada correctamente o si era clasificable. Por esto, las medidas de certeza pueden utilizarse para identificar imágenes para una revisión de expertos. Los análisis de bootstrapping indicaron más a fondo que el 90 % de las imágenes estuvieron clasificadas correctamente con sólo cinco voluntarios por imagen. Las clasificaciones de especies basadas en el voto de pluralidad de múltiples científicos ciudadanos puede proporcionar un fundamento fiable para un monitoreo a gran escala de la vida silvestre africana.

## Introduction

Modern citizen science, the engagement of the general public in the process of science, has enormous potential to expand the scope and scale of research in ecology and conservation. These fields have long benefited from volunteer contributions to, for example, the Audubon Society's Christmas Bird Count, which dates back more than 100 years (Silvertown 2009). In the last decade, technological advances have rapidly accelerated the number and diversity of projects that include public participation (Silvertown 2009; Dickinson et al. 2010, 2012; Tulloch et al. 2013).

Online projects engage people to contribute data on an extraordinary array of taxa around the world (e.g., Firefly Watch, HerpMapper, International Waterbird Census, and Road Watch) and on weather and climate (e.g., Citizen Weather Observer Program). Increased internet connectivity now allows volunteers to upload species sightings on websites such as iSpot.org and immediately interact with dozens of other naturalists (Silvertown et al. 2015). Integrating volunteer effort and emerging technologies expands the range of possibility in both basic and applied research.However, broad‐scale implementation of citizen science for research is hindered by concerns about data quality. Many professional researchers are skeptical of data produced by nonexperts, which lowers publication rates and grant funding of citizen science projects (Foster‐Smith & Evans 2003; Dickinson et al. 2010; Bonter & Cooper 2012). Although individual contributors can be measurably worse than trained professionals (Foster‐Smith & Evans 2003; Galloway et al. 2006; Delaney et al. 2007; Gardiner et al. 2012), solutions are available for assuring quality control of volunteer data.

Some projects train volunteers or require volunteers to pass a competency test, whereas others discard data from inexperienced or unreliable contributors (Dickinson et al. 2010). However, these procedures take time and may waste potentially valuable information and volunteer effort. Alternatively, eBird and FeederWatch ask volunteers to report bird sightings, flag implausible reports, and engage experts in validation of flagged contributions (Bonter & Cooper 2012). Although this approach can reduce false positives of unusual sightings, it leaves no way to verify plausible but erroneous entries.

Successful citizen science projects in astrophysics, such as Galaxy Zoo (Lintott et al. 2008; Willett et al. 2013), Space Warps (Marshall et al. 2016), Milky Way Project (Simpson et al. 2012; Beaumont et al. 2014), and Andromeda Project (Johnson et al. 2015) rely on the judgments of multiple volunteers to classify satellite and telescope imagery. Cyclone Center, a meteorological project, asks multiple users to identify features in infrared satellite images of storms (Hennon et al. 2014). Each of these projects applies algorithms to aggregate the responses and produces expert‐quality data sets. Similar approaches can be applied to wildlife and conservation‐based citizen science projects that ask volunteers to identify animals in photographs taken with camera traps.

Digital image collection from camera‐trap surveys is a rapidly expanding method (O'Connell et al. 2011) that is used to study rare and elusive species worldwide (e.g., Karanth & Nichols 2002; Dillon & Kelly 2007; Kelly et al. 2008) and to survey animals across large spatial extents (e.g., O'Brien et al. 2010; Kinnaird & O'Brien 2012; Bischof et al. 2014). However, as inexpensive digital cameras have proliferated, such surveys are increasingly limited by human processing capacity. Engaging citizen scientists to classify images can dramatically increase the amount of information researchers can extract from large data sets.

We devised an approach to produce accurate, reliable data from multiple untrained, nonexpert volunteers classifying images from the Snapshot Serengeti (www.snapshotserengeti.org) camera‐trapping study. We framed our analyses in terms of accuracy and efficiency to provide guidelines for optimizing the trade‐off between effort (of volunteers and experts) and accuracy. Because conservation studies often target rare species, we also evaluated how measures of accuracy and efficiency differed across species of contrasting rarity. Our overarching goal was to provide straightforward tools and quantifiable metrics to test and validate citizen science data, thus providing biologists with a generalizable approach for engaging citizen scientists to produce reliable data for large‐scale conservation and wildlife research.

## Methods

### The Snapshot Serengeti Interface

Snapshot Serengeti is hosted by the Zooniverse citizen science platform (www.zooniverse.org), which engages 1.5 million volunteers worldwide to participate in a broad array of projects. Zooniverse volunteers are motivated largely by a desire to contribute to science (Raddick et al. 2010, 2013) and engage with research teams and one another in high‐level scientific discussions via the Zooniverse discussion forums (Mugar et al. 2014).

On www.snapshotserengeti.org, members of the general public viewed and classified images from a large‐scale camera survey in Serengeti National Park, Tanzania (see Swanson et al. 2015 for survey details). From June 2010 to May 2013, the camera survey accumulated 99,241 camera‐trap days and produced 1.2 million image sets (each image set contained 1–3 images taken in a single burst over approximately 1 s). Within 3 d of launching the website, volunteers contributed 1 million species classifications and processed an 18‐month backlog of images (Swanson et al. 2015).

**Figure 1:**
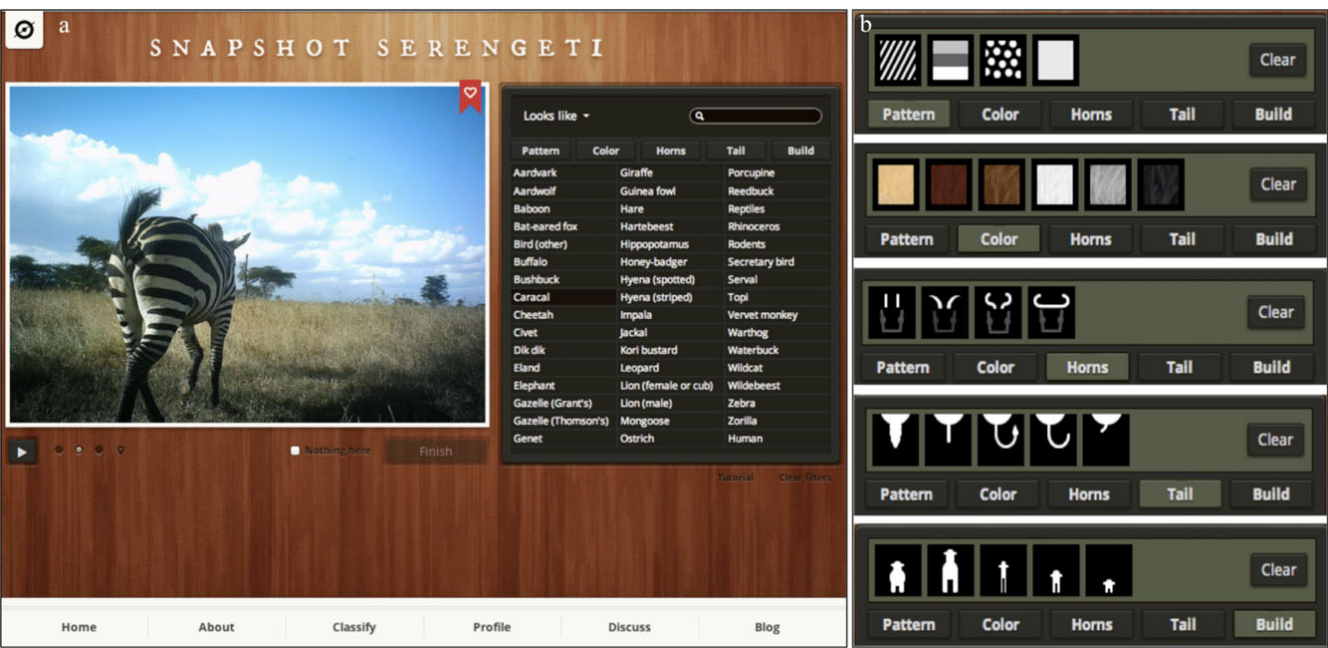
The Snapshot Serengeti website interface used for classifying species, counts, and behaviors in images from the camera‐trapping survey: (a) primary interface with all available species options and (b) filters that help narrow users’ choices when classifying species.

Users were asked to identify species, count the number of animals (binned as 1, 2, 3, 4, 5, 6, 7, 8, 9, 10, 11–50, and 51+ individuals), and characterize behaviors in each image set (Fig. 1). Volunteers followed a simple tutorial that explained the online interface, but they were not formally trained or tested for accuracy before contributing. We designed the interface to help guide people with no background knowledge through the process of animal identification from 48 possible species and taxonomic groups while providing a rapid route to classification for more knowledgeable participants. New users filtered potential species matches by morphological characteristics such as horn shape, body shape, color, pattern, tail shape, or a general gestalt (e.g., “looks like an antelope or deer”). More experienced users could select the species directly from a list. A “nothing here” button allowed users to classify images without any visible animals, but we did not provide an I‐don't‐know option because previous testing with this option with undergraduate volunteers on a small‐scale prototype indicated that such answers were overused and thus reduced the efficiency of volunteer contributions.

Each image was circulated to multiple users and retired after meeting the following criteria: the first 5 classifications were nothing here (hereafter blank); 10 nonconsecutive nothing‐here classifications (hereafter blank consensus); or 10 matching classifications of species or species combinations, not necessarily consecutive (hereafter consensus). If none of these criteria were met, the image was circulated until it accumulated 25 species classifications (hereafter complete). These values were chosen based on volunteer performance on existing Zooniverse projects. Volunteers classified Snapshot Serengeti data faster than images were produced, and images were recirculated for use in classrooms. As a result, the number of classifications for images containing animals ranged from 11 to 57 (mean = 26, median = 27).

### Data Aggregation and Validation

We implemented a simple plurality algorithm to transform the volunteer classifications for each image into a single aggregated classification. As described in Swanson et al. (2015), we first evaluated the median number, *n*, of different species reported by all classifiers for that image. For simplicity in interpreting volunteer accuracy, we limited our analyses here to the 94% of collected images with *n* = 1. We then identified the species present as the *n* species with the most classifications. For example, if an image with *n* = 1 had 15 total classifications, with 7 classifications of wildebeest, 5 classifications of buffalo, and 3 classification of topi, the aggregated classification would be wildebeest. If the same image had *n* = 2, the aggregated classification would be wildebeest and buffalo. We calculated the number of individuals present for each identified species by the median count (rounded up) of all raw classifications for that image and the interquartile range of counts reported by all classifiers for a given image.

We calculated 3 measures of certainty or confidence for each image: evenness, fraction blanks, and fraction support. Evenness was calculated from all classifications that were not blank for each image with Pielou's evenness index (Pielou 1966): $-(\sum_{i\;=\;1}^{S}p_{i}\ln p_{i})/\ln S$, where *S* is the number of different species reported by all volunteers and *p_i_* is the proportion of classifications received by species *i*. When all classifications were in agreement, we assigned a value of zero. The maximum value for this index is 1.0, indicating high disagreement among classifications. Fraction blank was calculated as the fraction of classifiers who reported nothing here for an image that was ultimately classified as containing an animal. Fraction support was calculated as the fraction of classifications that supported the aggregated answer (i.e., fraction support of 1.0 indicated unanimous support).

We compared overall plurality algorithm performance to a baseline expert‐verified data set of 3829 randomly sampled images. This data set (from Swanson et al. 2015) was produced by asking a panel of experts to review these images. The experts were individuals who had undergone extensive formal training, passed qualification exams, or had years of experience identifying African wildlife. Of these images, 135 were independently classified by >1 expert. In cases where experts disagreed with the results of the plurality algorithm or had marked an image set as particularly difficult or impossible, A.S. and C.P. made the final authoritative species identification. For species‐specific analyses, we used an expanded data set of 5558 images that included extensive random sampling of images identified as rare species by the plurality algorithm to ensure their adequate representation (species‐specific sample sizes are in Supporting Information). In 0.8% of images, the panel of experts agreed that no authoritative species identification could be made. Because the citizen science interface does not allow for an impossible classification, the aggregated answers were technically wrong for these images because no reliable volunteer answer exists. Additional details of the field study, classification interface, aggregation, and validation are available in Swanson et al. (2015).

### Accuracy

We compared the results of the plurality algorithm with expert answers for species identification and animal counts. We evaluated overall algorithm accuracy by calculating the proportion of times the aggregated answer for species present was confirmed by expert classifications (reported as proportion correct) in the baseline expert‐validated data set. We calculated accuracy both for the resolvable images and for all images. We calculated species‐specific accuracy as the likelihood of the aggregated answer being correct (i.e., the likelihood of the expert classifications confirming the given aggregated answer) based on images in the expanded expert‐verified data set.We further evaluated species‐specific accuracy with respect to 2 types of error: false positives (species reported when not present) and false negatives (species not reported when actually present). Because the false‐negative analysis required images to be randomly sampled with respect to the true answer, this analysis was limited to the baseline expert‐verified data. The false‐positive analysis was applied to the expanded data set because it only required that images be randomly sampled with respect to the aggregated answer. For each species, we evaluated the proportion of photographs containing each type of error. We associated a group of species that were easy to identify with zero error, and then compared nonzero error rates to species commonness (defined as the total number of times a species was photographed) with simple linear regression on log‐transformed variables on the remaining species.

To assess the accuracy of counts, we first evaluated the agreement among expert counts for the 135 images with multiple expert classifications. We also calculated count ranges for each image from the raw classifications and compared expert agreement with these ranges. We calculated count range as the interquartile range (i.e., 25th percentile to 75th percentile values) of counts reported by all classifiers for a given image. We evaluated how often expert counts fell within this range for all resolvable images in the baseline expert‐verified data set. To assess image difficulty for effectively targeting expert review, we evaluated the 3 certainty measures against accuracy, classifying images as correct (plurality answer agreed with expert‐verified data set), incorrect (plurality answer disagreed with expert‐verified data set), or impossible (experts could not identify the species). To test the predictive power of these metrics, we performed a one‐way analysis of variance (ANOVA) followed by post‐hoc Tukey test (package stats in Program R) for each metric to evaluate whether mean evenness, fraction support, or fraction blanks differed significantly across correct (*n* = 5281), incorrect (*n* = 225), and impossible (*n* = 52) images from the extended expert‐verified data set. We further used a simple linear regression of log‐transformed mean species‐specific values of evenness, fraction support, and fraction blanks on the logarithm of the total number of times a given species was photographed to assess the effects of species commonness on the 3 measures of difficulty.

### Efficiency

We determined how many volunteer classifiers were needed to produce reliable species identifications by bootstrapping the plurality answer from raw classifications for images with expert‐verified answers. We first excluded the expert answers from the raw data set. Then, for every image, we randomly sampled (with replacement) *n* classifications from the raw data set (20 iterations each) and applied the plurality algorithm to produce an aggregated answer and calculate an evenness score. In case of ties, one answer was randomly selected from the 2.

For each *n* volunteers, we evaluated accuracy as the proportion of times the plurality algorithm agreed with the expert‐verified answer. Overall accuracy was calculated using images from the expert‐verified data set. We further characterized species as belonging to 1 of 3 groups: high overall accuracy (i.e., low false‐positive and false‐negative rates), high false positives, and high false negatives. We calculated species‐specific accuracy for species in each of these groups. Species‐specific accuracy was calculated for the expanded data set as the probability of the aggregated answer being correct.

Because images vary in identification difficulty, we further evaluated the number of classifiers needed to provide an evenness score that reliably indicated a correct answer. For every iteration of the bootstrapping analysis for every *n* classifications, we calculated the evenness score for a given image. At every additional classification, we split images into those with evenness scores of <0.5 and >0.5 and calculated the proportion of images in each group (<0.5 and >0.5) that were correct.

## Results

### Accuracy

**Figure 2:**
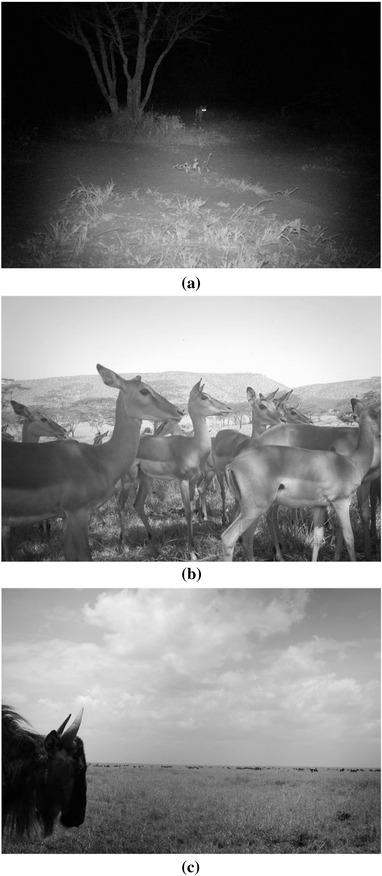
Example images from the Serengeti camera survey and presented on the Snapshot Serengeti website illustrating situations in which: (a) species identification is impossible, (b) a precise count of animals is impossible, and (c) animals present in foreground and background that leads to a wide range of individual counts by volunteers.

Experts identified species in 3800 of 3829 randomly sampled images (99.2%), labeling 29 images as impossible or unresolvable (e.g., Fig. 2a). The plurality algorithm agreed with experts on 3750 images, yielding 97.9% overall agreement and 98.6% agreement on resolvable images. Accuracy differed dramatically by species, ranging from 100% accuracy for giraffe (*n* = 87) and hippopotamus (*n* = 28) to 70% for jackals and 50% for aardwolves (see full list in Supporting Information).

**Figure 3:**
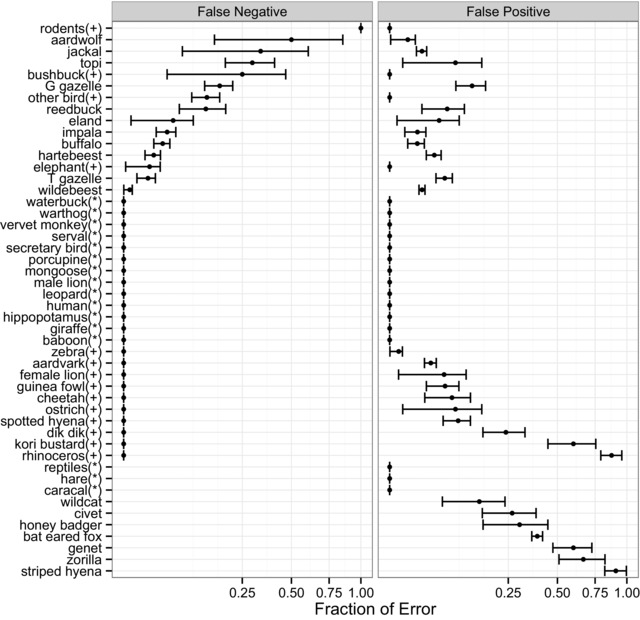
Species‐specific rates of false‐negative (calculated relative to the randomly sampled expert‐verified data set of 3829 images) and false‐positive (calculated relative to the extended expert‐verified data set of 5558 images) error for species identifications produced by the plurality algorithm (error bars, standard error calculated for proportions; *, species with zero total error; +, species with zero false‐positive error). Note that x‐axis is plotted on a square‐root scale. Sample sizes are in Supporting Information.

**Figure 4:**
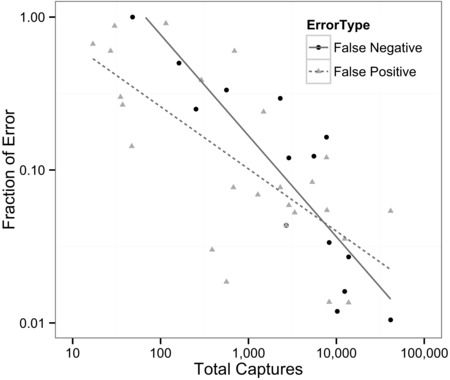
False‐positive and false‐negative (log fraction of error) identification of animals versus species commonness (frequency of species appearance in the overall data set, given as log[total number of pictures]). Linear regression was performed on log‐log transformed variables and restricted to species with nonzero error rates. Species with an asterisk in Fig. 3 were excluded from false‐positive and false‐negative analyses, and species in Fig. 3 marked with a plus were excluded from the false‐positive analysis.

Species‐specific accuracy also varied in the type of error (Fig. 3): species with high false negatives tended to have fewer false positives. A few species had high rates of both false positives and negatives, and these tended to be confused with each other. Confusion was typically clustered within groups of species with similar sizes, shapes, and colors (Supporting Information). Algorithm accuracy was generally related to rarity. Species with fewer photographs had higher rates of false negatives and false positives (Fig. 4), although a subset of species were perfectly classified regardless of how often they appeared in the data set (Fig. 3). Both error types were significantly higher for rare species, although stronger for false negatives (*p* ≤ 0.0001, *r* ^2^ = 0.71, df = 13) than for false positives (≤0.0001, *r* ^2^ = 0.45, df = 26).

Precise counts were unresolvable in many images (e.g., Fig. 2b). Experts agreed on the number of individuals only 74% of the time (of 135 photos), and the average range of expert answers spanned 2.5 bins. The median count reported by the plurality algorithm was just as likely to agree with experts as experts were to agree among themselves (75.2% of 3800 images).

The interquartile range reported by the plurality algorithm was generally precise. Volunteers agreed on a single count in 50% of images, and 86% of images had ranges of ≤3 bins (e.g., 4–6 animals) (Supporting Information). In images with a wide range of counts, animals appeared in both the foreground and background (Fig. 2c), and the distribution of counts tended to be bimodal (Supporting Information), presumably due to some users only counting animals in the foreground and others counting everything in the image.

**Figure 5:**
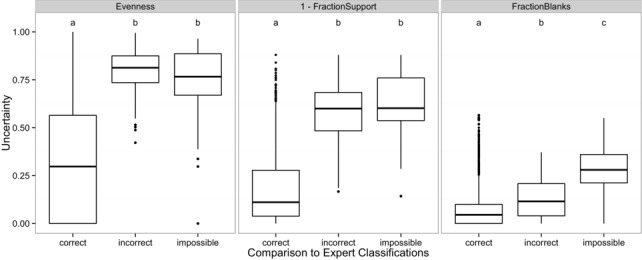
Evenness (support level of agreement among classifications), fraction blanks (fraction of classifiers who reported “nothing here”), and fraction support (fraction of classifications supporting the aggregated answer) for images that were verified by experts and deemed to be correct (aggregated volunteer answer agreed with expert answer), incorrect (aggregated volunteer answer did not agree with expert answer), or impossible (experts could not determine the species present). All metrics are bounded between 0 and 1, fraction support is plotted as “1‐fraction support” so that for all 3 metrics, scores closer to 1 reflect greater uncertainty. Boxplots marked with *(a)* have significantly different means than those marked with *(b)*.

Evenness, fraction support, and fraction blank were all excellent predictors of whether aggregated answers were likely to be correct (Fig. 5). The post‐hoc Tukey test revealed that evenness scores were significantly lower (i.e., answers were all skewed toward a single species) for images that were classified correctly than for images that were incorrectly classified (*p* < 0.0001) or impossible (*p* < 0.0001). Similarly, fraction support was higher for images that were correctly identified than for images that were incorrectly classified (*p* < 0.0001) or impossible to classify (*p* < 0.0001). However, evenness stood out as the single best predictor of a correct classification: 99.9% of images with evenness < 0.25 were correct and 99.8% of images with evenness < 0.5 were correct. In contrast, only 85% of images with evenness > 0.5 and 71% of images with evenness > 0.75 were correct.

Although evenness (*p* = 0.157) and fraction support (*p* = 0.394) for incorrect and impossible images did not differ significantly, the fraction of nothing‐here classifications differed significantly across all 3 groups, and fraction blank was the best predictor of whether an image was ultimately resolvable (*p* < 0.0001 for all pairwise comparisons). Images that evoked a large proportion of nothing‐here responses sometimes contained a partial or blurred view of an animal, making it particularly difficult to identify. Thus, some classifiers apparently preferred to state there was "nothing here" rather than to guess the animal's identity.

The vast majority of images were classified as easy—showing high levels of agreement on species classification (Supporting Information). For example, half of all images had >87% agreement on the final answer, and only 6% of images did not attain a majority (i.e., no species had >50% of the classifications). Excluding classifications of nothing here, 36% of images had unanimous agreement on the species classification. As with accuracy, rare species were significantly more difficult to identify than common species: regression of certainty scores versus log of total pictures showed higher fraction support (*p* < 0.0001, *r* ^2^ = 0.397), lower evenness (*p* < 0.001, *r* ^2^ = 0.214, df = 46), and lower fraction blanks (*p* = 0.0036, *r* ^2^ = 0.17, df = 46) with increasing commonness (Supporting Information).

### Efficiency

**Figure 6:**
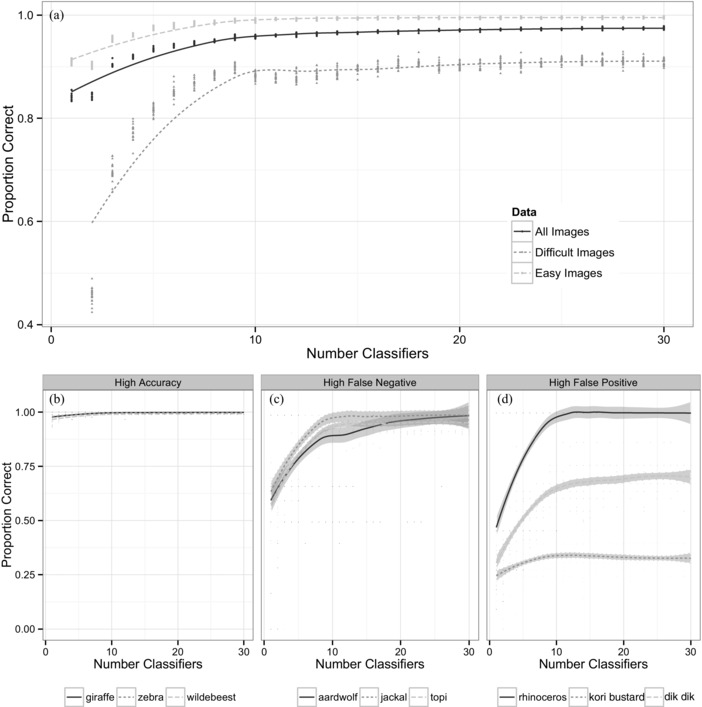
(a) Accuracy of bootstrapped plurality algorithm applied to volunteer classification (20 iterations) for each *n* volunteer species identifications. Proportion correct is the proportion of times the expert classifications agreed with the aggregated volunteer answer for resolvable images (i.e., images in which the expert could determine the species present) (solid black line, accuracy calculated for all images; dark gray dashed line, accuracy for images characterized as difficult [evenness >0.5], and light gray dashed line, accuracy for images characterized as easy [evenness ≤0.5], where evenness scores were calculated dynamically after every additional classification). Species characterized by (b) high accuracy, (c) high false‐negative identifications, and (d) high false‐positive identifications.

Accuracy increased asymptotically with the number of volunteers (Fig. 6a). Overall, we achieved 90% accuracy on species identifications with 5 volunteer classifications and 95% accuracy with 10 classifications.

The number of volunteer classifications needed for accurate answers differed with species (Fig. 6b). Images with easy species (characterized by low rates of false negatives and false positives) were nearly always correct with just 1–3 classifiers. Images with high rates of false negatives (e.g., jackals, aardwolves, and topi) needed more classifiers to achieve high accuracy rates, but additional improvement declined after about 10 classifications. In contrast, for species with high false‐positive rates (such as the extremely rare civets, genets, and striped hyenas), accuracy rates remained low even after 30 classifiers.

Evenness scores were excellent predictors of an accurate classification (Fig. 6a & Supporting Information). After 3 classifications, images with evenness ≤0.5 (e.g., at least 2 of the 3 users agreed on the species) were ≥97% likely to be correct. After 5 classifications, images with an evenness score of ≤0.5 were ≥99% likely to be correct. Thus, evenness can quickly be used to identify images requiring additional classifications.

## Discussion

Snapshot Serengeti provides a case study in engaging citizen scientists in rapidly and accurately processing large volumes of ecological imagery. Unlike many other citizen science projects (Dickinson et al. 2010), Snapshot Serengeti volunteers were neither trained nor required to demonstrate species identification skills. Instead, we engaged multiple volunteers for every task and aggregated their answers to produce highly accurate data. Snapshot Serengeti data were 97.9% accurate overall, whereas 85–95% accuracy is reported for projects engaging trained volunteers (Galloway et al. 2006; Delaney et al. 2007; Gardiner et al. 2012). Engaging multiple volunteers for every image did not necessarily mean many volunteers for every image. By evaluating measures of certainty in volunteer answers and evaluating the relative contribution of additional volunteer classifications, we provide guidelines for researchers to target volunteer effort and expert effort to balance their data needs and available human resources for specific projects.

### Accuracy

Aggregating multiple answers was critical to producing the high rates of accuracy on Snapshot Serengeti. Individual volunteers demonstrated similar levels of accuracy as volunteers for other projects (approximately 85% [e.g., Dickinson et al. 2010]), and accuracy differed with experience (Supporting Information).

Having multiple people classify an image was more reliable than a single person—even when that single person was an expert. The aggregated volunteer answers were even more accurate (97.9%) than those of individual experts (96.6%) when compared with the consensus expert assessments. Experts can make mistakes: a single field researcher flipping through hundreds or thousands of photographs can become fatigued and miss species or click on the wrong classification. Making a precise count was impossible in many images (Fig. 2b). Calculating a count range from multiple volunteers provided more reliable count data than a single number reported by a single expert (recall that experts disagreed on counts for 26% of images).

When creating the expert‐verified data, experts agreed that a small number of photographs (0.8%) were impossible to classify (Fig. 2a). These impossible photographs accounted for 36% of the overall error because the Snapshot Serengeti interface did not allow users to mark images as such. However, the likelihood of an image truly being impossible to identify can be determined by the fraction of blanks reported in volunteer classifications. Furthermore, even guesses provide information, such as distinguishing between a small nocturnal insectivore and a large ungulate.

As found in many other projects, citizen scientist accuracy differed by species (Dickinson et al. 2010). A subset of species was perfectly classified regardless of how many times they appeared in the data set. These species tended to be visually striking (e.g., giraffe, porcupine, male lion, and waterbuck) and thus clearly identifiable even to inexperienced volunteers. In contrast, rare species had higher rates of both false‐positive and false‐negative errors (Fig. 3), mirroring results from other studies. Rare species present fewer opportunities for learning, and people are especially eager to report rare or unique species (Galloway et al. 2006; Delaney et al. 2007).

False positives and false negatives have different implications for conservation research. False positives are typically calculated and corrected in citizen science projects through expert review or detectors meant to catch unlikely classifications. False negatives (failure to identify an animal that is present) are often implicitly assumed by camera‐trap projects to arise exclusively from failure to photograph animals (MacKenzie et al. 2002, 2006; Royle & Dorazio 2008). However, we found that false negatives were also caused by a failure of classifiers to identify an animal, which can be especially problematic when studying rare and elusive species.

The lack of false‐negative detection is a critical limitation of existing validation protocols in projects that flag implausible sightings for expert review but overlook plausible errors, such as misidentifying a rare species as a common one (Bonter & Cooper 2012). The multiple‐volunteer approach specifically addresses this limitation by providing simple metrics that reflect the likelihood of an image being correctly classified (Fig. 5). Because errors tend to be clustered between species of similar morphology (Supporting Information), false negatives can be addressed by reviewing all images with low certainty scores reported as an animal similar in appearance to the target species.

Evenness and fraction support both provide simple, reliable metrics that reflect the likelihood of an image being correctly identified. Researchers can set threshold values below which images can be targeted for review or exclusion. The volume of images for expert review depends on the certainty threshold required by the research questions as well as the frequency of the target species in the data set.

### Efficiency

For projects that are limited by classifier effort, minimizing per‐image effort is critical to timely data processing. Maximizing efficiency requires balancing volunteer effort, expert effort, and levels of acceptable error for a particular analysis.

In Snapshot Serengeti, images achieved approximately 90% accuracy at 5 classifiers, 95% accuracy at 10 classifiers, and asymptotically approached 98% accuracy after 20 classifiers (Fig. 6a). Efficiency differed according to species (Fig. 6b). Whereas common or easily recognizable species were almost always correctly classified with 2 or 3 volunteers, rare species sometimes needed 10 or more classifiers to achieve similar levels of accuracy. For some rare species, accuracy rates improved only negligibly with additional classifiers.

For a study focusing on relatively rare species, even small rates of error could have a substantial effect on results. Thus, an expert should inspect all images with evenness scores of >0.5 that have been identified as the target species (to eliminate false positives) and images with morphologically similar species (to eliminate false negatives). Adopting a less cautious threshold (say, evenness >0.75) would reduce the number of images to review.

Certainty metrics can be used dynamically to assess whether an image needs additional volunteer classifications. The evenness metric quickly becomes a reliable indicator of whether an image is likely to be correct or incorrect: with 2 volunteers, 90% of images with evenness <0.5 are correct, and with 5 volunteers, 97% of images with evenness <0.5 are correct. The decision to send an image to more volunteers, flag it for expert review, or exclude it from analysis ultimately depends on the needs and resources of a given project.

With the widespread engagement of the general public in scientific research, projects must carefully consider how best to produce accurate, reliable data (Tulloch et al. 2013; Wiggins et al. 2014). Based on lessons from Snapshot Serengeti, we recommend the following. First, engage multiple classifiers for each image and calculate an aggregated answer from multiple classifications. Sending an image to 10 volunteers, instead of 1, increased accuracy from 85% to 95%. Second, do not allow answers, such as I don't know or impossible, that lack information about a correct classification. The variation in allowable answers can indicate whether an image is likely to be difficult or impossible. Third, produce expert‐verified data to validate aggregated classifications and to measure certainty for every project. Accuracy should be assessed on a per‐project basis because it depends on the ecological system, taxa, volunteer interface, and the volunteer base. Fourth, balance effort between experts and volunteers according to project needs and capacity. Set baseline levels of accuracy, determine the necessary number of volunteer classifications, and estimate the effort that must be devoted by experts to review difficult or ambiguous images.

Engaging multiple citizen scientists in image classification is not limited to remote camera surveys. Many current projects ask people to essentially collect and analyze data on the spot (e.g., identifying animals or plants seen and submitting a written record). Volunteers could instead collect data as photographs that can then be analyzed by multiple volunteers for validation, thereby increasing quality of the data.

A multiple‐classifier approach to engaging citizen science has dramatic implications for ecology and conservation biology—both for basic and applied research. For example, in conservation monitoring, single researchers or small teams often deploy camera traps to study specifically a single rare species (Karanth 1995; Dillon & Kelly 2008; O'Connell et al. 2011). To keep pace with high‐volume data production, conservationists often resort to classifying only those images containing their target species—discarding enormous amounts of image data that could otherwise be used for multispecies monitoring. By engaging citizen scientists in the processing of every image and limiting expert review to only those photos with low certainty scores, multispecies camera surveys could dramatically expand the scope of conservation monitoring.

## Supporting information

Confusion matrix for species identifications (Appendix S1), precision of volunteer‐contributed animal counts (Appendix S2), distribution of volunteer counts for images with large count ranges (Appendix S3), distribution of certainty metrics (Appendix S4), volunteer accuracy compared to number of contributions (Appendix S5), species‐specific sample size and error rates (Appendix S5), and ANOVA results for certainty measures as predictors of whether images were classified correctly, incorrectly, or were impossible to classify (Appendix S6) are available online. The authors are solely responsible for the content and functionality of these materials. Queries (other than absence of the material) should be directed to the corresponding author.
