# Supplementary material for: A generalized approach for producing, quantifying, and validating citizen science data from wildlife images
Source: Conserv Biol. 2016 Apr 25;30(3):520–31. doi: 10.1111/cobi.12695 (PMC4999033; doi:10.1111/cobi.12695)
Supplement: Supplementary file 1 — Confusion matrix for species identifications (Appendix S1), precision of volunteer‐contributed animal counts (Appendix S2), distribution of volunteer counts for images with large count ranges (Appendix S3), distribution of certainty metrics (Appendix S4), volunteer accuracy compared to number of contributions (Appendix S5), species‐specific sample size and error rates (Appendix S5), and ANOVA results for certainty measures as predictors of whether images were classified correctly, incorrectly, or were impossible to classify (Appendix S6) are available online. The authors are solely responsible for the content and functionality of these materials. Queries (other than absence of the material) should be directed to the corresponding author. [file COBI-30-520-s001.docx]

**SUPPLEMENTARY MATERIALS**

**Appendix 1**

Confusion matrix for all species in the randomly sampled baseline gold-standard dataset. Consensus species identification is given on the y-axis; gold standard classification on the x-axis. Color of grid cells reflects the percent of time that any given species was classified as any species. Diagonal line reflects when species were correctly identified. For example, Zebra were correctly identified 100% of the time. In contrast, reedbuck were sometimes identified as dik dik or Thomsons’s gazelle, but neither dik dik nor Thomson’s were identified as reedbuck.

**Appendix 2**

Cumulative distribution of the spread for count ranges. Count precision is calculated as the difference between the minimum and maximum counts within 50^th^ percentile range (e.g. 75^th^ percentile count – 25^th^ percentile count).

**Appendix 3**

Distribution of counts for images with count range > 10. Most volunteers reported either one animal or more than 10 animals. This distribution suggests that volunteers are inconsistent in whether they report only foreground animals or both foreground and background animals. This inconsistency stems from lack of clear instructions on the Snapshot Serengeti interface.

**Appendix 4**

Distribution of evenness, fraction support, and fraction blanks across all images.

**Appendix 5:** Average accuracy plotted against the number of images classified. We extracted raw classifications of all logged-in volunteers for resolvable images in the baseline gold-standard dataset (3,800 images, 5,833 volunteers, 91,140 classifications). For each volunteer we calculated the total number of images classified and average accuracy across all images. We then binned volunteers into groups by rounding number of classifications to the nearest 10 and ran a simple linear regression of average accuracy vs. number of images classified. Median individual accuracy was 88.8% correct, and accuracy was higher among volunteers who classified more images (p < 0.0001, r^2^ = 52.7, df = 61)

**Appendix 6:** Mean evenness (support level of agreement among classifications), fraction blanks (fraction of classifiers who reported “nothing here”), and fraction support (fraction of classifications supporting the aggregated answer), for each species (calculated from the aggregated volunteer identifications) plotted against species commonness, given as log total number of pictures.

**Appendix 6** Number of images for all species in overall dataset, the baseline randomly sampled gold standard dataset, and the extended gold standard dataset in which species reported as rare species were extensively sampled to expand the dataset. Accuracy is the probability of gold standard data confirming the consensus answer for a species. False Negative rates are calculated for baseline gold standard data; false positives are calculated for the extended gold standard data.

| **Species** | **Overall (n)** | **Baseline Gold Standard (n)** | | **Extended Gold Standard (n)** | **Accuracy** | | **False negatives** | **False positives** |
| --- | --- | --- | --- | --- | --- | --- | --- | --- |
| aardvark | 386 | 4 | 399 | | | 0.970 | 0.000 | 0.030 |
| aardwolf | 162 | 1 | 171 | | | 0.994 | 0.500 | 0.006 |
| baboon | 1,557 | 22 | 28 | | | 1.000 | 0.000 | 0.000 |
| batEaredFox | 291 | 1 | 297 | | | 0.613 | NA | 0.387 |
| buffalo | 13,779 | 219 | 221 | | | 0.986 | 0.027 | 0.014 |
| bushbuck | 253 | 3 | 17 | | | 1.000 | 0.250 | 0.000 |
| caracal | 79 | - | 15 | | | 1.000 | NA | 0.000 |
| cheetah | 1,279 | 6 | 29 | | | 0.931 | 0.000 | 0.069 |
| civet | 37 | - | 15 | | | 0.733 | NA | 0.267 |
| dikDik | 1,485 | 10 | 25 | | | 0.760 | 0.000 | 0.240 |
| eland | 2,690 | 23 | 23 | | | 0.957 | 0.043 | 0.043 |
| elephant | 10,242 | 83 | 83 | | | 1.000 | 0.012 | 0.000 |
| gazelleGrants | 7,724 | 58 | 58 | | | 0.879 | 0.164 | 0.121 |
| gazelleThomsons | 41,424 | 200 | 204 | | | 0.946 | 0.010 | 0.054 |
| genet | 27 | - | 15 | | | 0.400 | NA | 0.600 |
| giraffe | 8,395 | 87 | 87 | | | 1.000 | 0.000 | 0.000 |
| guineaFowl | 7,807 | 55 | 55 | | | 0.945 | 0.000 | 0.055 |
| hare | 398 | - | 15 | | | 1.000 | NA | 0.000 |
| hartebeest | 12,435 | 254 | 254 | | | 0.965 | 0.016 | 0.035 |
| hippopotamus | 2,614 | 28 | 28 | | | 1.000 | 0.000 | 0.000 |
| honeyBadger | 35 | - | 10 | | | 0.700 | NA | 0.300 |
| human | 9,869 | 70 | 70 | | | 1.000 | 0.000 | 0.000 |
| hyenaSpotted | 5,319 | 55 | 72 | | | 0.917 | 0.000 | 0.083 |
| hyenaStriped | 115 | 1 | 11 | | | 0.091 | NA | 0.909 |
| impala | 8,287 | 146 | 146 | | | 0.986 | 0.034 | 0.014 |
| jackal | 562 | 2 | 539 | | | 0.981 | 0.333 | 0.019 |
| koriBustard | 693 | 10 | 10 | | | 0.400 | 0.000 | 0.600 |
| leopard | 228 | 3 | 8 | | | 1.000 | 0.000 | 0.000 |
| lionFemale | 3,359 | 18 | 19 | | | 0.947 | 0.000 | 0.053 |
| lionMale | 923 | 1 | 10 | | | 1.000 | 0.000 | 0.000 |
| mongoose | 247 | 4 | 4 | | | 1.000 | 0.000 | 0.000 |
| ostrich | 674 | 3 | 13 | | | 0.923 | 0.000 | 0.077 |
| otherBird | 5,552 | 57 | 59 | | | 1.000 | 0.123 | 0.000 |
| porcupine | 289 | 8 | 18 | | | 1.000 | 0.000 | 0.000 |
| reedbuck | 2,879 | 24 | 34 | | | 0.941 | 0.120 | 0.059 |
| reptiles | 131 | - | 10 | | | 1.000 | NA | 0.000 |
| rhinoceros | 30 | 1 | 16 | | | 0.125 | 0.000 | 0.875 |
| rodents | 48 | - | 10 | | | 1.000 | 1.000 | 0.000 |
| secretaryBird | 435 | 4 | 18 | | | 1.000 | 0.000 | 0.000 |
| serval | 462 | 6 | 20 | | | 1.000 | 0.000 | 0.000 |
| topi | 2,300 | 13 | 13 | | | 0.923 | 0.294 | 0.077 |
| vervetMonkey | 314 | 1 | 15 | | | 1.000 | 0.000 | 0.000 |
| warthog | 7,512 | 112 | 112 | | | 1.000 | 0.000 | 0.000 |
| waterbuck | 354 | 1 | 14 | | | 1.000 | 0.000 | 0.000 |
| wildcat | 47 | - | 14 | | | 0.857 | NA | 0.143 |
| wildebeest | 100,968 | 1,550 | 1,552 | | | 0.981 | 0.001 | 0.019 |
| zebra | 70,650 | 685 | 693 | | | 0.999 | 0.000 | 0.001 |
| zorilla | 17 | - | 9 | | | 0.333 | NA | 0.667 |

**Table S2**: Results from one-way ANOVA tests for differences in mean evenness, fraction support, and fraction blanks for images that were classified correctly, incorrectly, or were determined to be impossible by experts.

|  |  |  |  |  |  |
| --- | --- | --- | --- | --- | --- |
| **Response: Evenness** | **Df** | **Sum Sq** | **Mean Sq** | **F value** | **Pr(>F)** |
| Correct/Incorrect/Impossible | 2 | 58.79 | 29.3945 | 350.76 | 2.20E-16 |
| Residuals | 5555 | 465.52 | 0.0838 |  |  |
|  |  |  |  |  |  |
| **Response: FractionSupport** | **Df** | **Sum Sq** | **Mean Sq** | **F value** | **Pr(>F)** |
| Correct/Incorrect/Impossible | 2 | 44.052 | 22.0261 | 689.66 | 2.20E-16 |
| Residuals | 5555 | 177.413 | 0.0319 |  |  |
|  |  |  |  |  |  |
| **Response: FractionBlanks** | **Df** | **Sum Sq** | **Mean Sq** | **F value** | **Pr(>F)** |
| Correct/Incorrect/Impossible | 2 | 2.773 | 1.38643 | 164.92 | 2.20E-16 |
| Residuals | 5555 | 46.699 | 0.00841 |  |  |
|  |  |  |  |  |  |
|  |  |  |  |  |  |
